# Comprehensive analysis of pyroptosis regulation patterns and their influence on tumor immune microenvironment and patient prognosis in glioma

**DOI:** 10.1007/s12672-022-00474-5

**Published:** 2022-03-10

**Authors:** Tianyu Fan, Yi Wan, Delei Niu, Bin Wang, Bei Zhang, Zugui Zhang, Yue Zhang, Zheng Gong, Li Zhang

**Affiliations:** 1 The Department of Immunology, School of Basic Medicine, Qingdao University, 308 Ningxia Road, Qingdao, Shandong China; 2 The Department of Pathogenic Biology, School of Basic Medicine, Qingdao University, 308 Ningxia Road, Qingdao, Shandong China; 3 Institute for Research on Equity and Community Health, Christiana Care Health System, Newark, USA; 4 Sino-Cellbiomed Institutes of Medical Cell & Pharmaceutical Proteins Qingdao University, 308 Ningxia Road, Qingdao, Shandong China; 5 Department of Basic Medicine, Xiangnan University, 889 Chenzhou Avenue, Hunan Chenzhou, China

**Keywords:** Glioma, Pyroptosis, Immune, Prognosis

## Abstract

**Background:**

Glioma is the most common intracranial malignancy with a poor prognosis. Although remarkable advances have been made in the study of diagnostic and prognostic biomarkers, the efficacy of current treatment strategies is still unsatisfactory. Therefore, developing novel and reliable targets is desperately needed for glioma patients. Pyroptosis reshapes tumor immune microenvironment (TME) and promotes the destruction of the tumor by the immune system. Moreover, pyroptosis levels correlate with prognosis and immunotherapy response in many cancer patients. This study performed a comprehensive analysis of pyroptosis in the glioma, unveiling its potential value in glioma prognosis prediction and therapy efficacy.

**Methods:**

Firstly, the pyroptosis regulation patterns were comprehensively evaluated on 33 pyroptosis-related genes in 1716 glioma samples. The correlations were analyzed between pyroptosis regulation patterns and TME immune cell infiltration properties. Next, pyroptosis regulation patterns were measured by the PSscore model based on principal component analysis algorithms. The correlations were analyzed between PSscore and tumor mutational burden (TMB), immune checkpoint blockade (ICB) therapeutic advantages. Last, the findings were validated in an independently collected external clinical cohort.

**Results:**

We determined two distinct pyroptosis regulation patterns. The cluster-A was high immune cell infiltration with a poor prognosis (*p* < 0.001), whereas the cluster-B was low immune cell infiltration with a better prognosis (*p* < 0.001). We developed the PSscore as a measure for pyroptosis regulation patterns. The high PSscore with an inflamed TME phenotype, a high TMB (*p* < 0.0001), increased innate immune response, and a poor prognosis (*p* < 0.001). It was in stark contrast to the low PSscore (*p* < 0.001). Analysis of PSscore with checkpoint therapy indicated high PSscore were correlated with enhanced response to anti-PD-1 immunotherapy (*p* = 0.0046). For validation, we utilized in vitro experiments on an external clinical cohort. The results demonstrated that GSDMD expression level in the high PSscore group was significantly upregulated compared to the low PSscore group (*p* < 0.001); the CD3+ T cells and the CD3+PD-1+ cells significantly increased in the high PSscore group compared to the low PSscore group (*p* < 0.01).

**Conclusions:**

The PSscore of pyroptosis regulation pattern is a reliable biomarker, and it is valuable to predict prognosis, TME, and ICB therapeutic efficiency in glioma patients.

**Supplementary Information:**

The online version contains supplementary material available at 10.1007/s12672-022-00474-5.

## Introduction

Glioma is the most common intracranial malignancy [1]. Over the past four decades, various therapies for glioma have been developed. However, the prognosis of glioma is still poor and far from satisfactory. In recent years, significant progress has been made to identify molecular markers that could predict the glioma’s progression and clinical prognosis, such as IDH1, 1p19q, and MGMT methylation [2–4]. However, these parameters are not ideal due to their limited accuracy, sensitivity, and specificity. Therefore, there is an urgent requirement for feasible and reliable indicators to predict the clinical outcomes following therapy, especially in immunotherapy.

Pyroptosis is an inflammatory programmed cell death pathway resulting in cell swelling with membrane rupture and ultimately cell death. A series of pro-inflammatory mediators are released during this process, including interleukin (IL)-1β and IL-18. They promoted immune response and altered the tumor’s immune microenvironment [5–8]. One such protein family is GSDM which plays an essential role in pyroptosis by mediating inflammation and cell death. It consists of gasdermin A (GSDMA), gasdermin B (GSDMB), gasdermin C (GSDMC), gasdermin D (GSDMD), gasdermin E (GSDME), and DFNB59 [9–12]. In recent years, the role of pyroptosis in tumors has become increasingly prominent. Studies have shown that cell pyroptosis may play a double-edged role in the occurrence and treatment of tumors. On one aspect, inflammatory molecules released by pyroptosis may induce the transformation of normal cells into tumor cells [13]. On the other one, tumor antigens and cytokines are released from the tumor and contribute to modulating tumor microenvironment (TME) and inhibiting the development of tumors [14]. Moreover, studies indicate pyroptosis contributed to destroying the tumor in cancer immunotherapy through modifying the TME, implying its undoubted role as a new target for glioma therapy [15–18]. In addition, it is worth noting that only some patients with immune checkpoint blockade therapy experience durable responses (anti-PD-1/L1 and anti-CTLA-4), and the large majority of patients do not meet a clinical need or experience any clinical benefit [19]. And pyroptosis may play an important role in evaluating the efficacy of checkpoint therapy via the characterization of tumor immune infiltration [20, 21]. However, up to date, the detailed role of pyroptosis in glioma has not been fully elucidated. Therefore, urgently need to answer: which ones consist of the pyroptosis pattern in TME? How does the pyroptosis pattern affect immune infiltration? How construct a reliable model based on the pyroptosis pattern to predict prognosis and the outcome of immunotherapy?

In the present study, we comprehensively evaluated the correlation between pyroptosis regulation patterns and TME cell-infiltrating characteristics via integrating the data of 1716 glioma samples from CGGA and TCGA databases. Two different pyroptosis regulation patterns, three different gene clusters with the TME characteristics, and nonnegative matrix factorization (NMF) clustering were identified. We constructed a scoring scheme (PSscore) that quantified pyroptosis regulation patterns to predict a patient’s prognosis by overall survival (OS) and outcome of ICB therapy. Finally, the results were verified in independently collected clinical gliomas.

## Methods

### Source and preprocessing of glioma databases

Gene expression data (FPKM values) of glioma samples were searched from publicly available data sets of the TCGA (https://portal.gdc.cancer.gov) and CGGA (www.cgga.org.cn). The matched normal data from 204 GTEx (https://commonfund.nih.gov/GTEx/) and five matched normal TCGA samples as a control. In Table S2, we summarized the information of all useful glioma and normal datasets.

### Unsupervised clustering for pyroptosis-related genes

In total, 33 related genes were extracted from pyroptosis research literature [13, 14, 22–24] (Table S3). Based on the expression of 33 pyroptosis-related genes, unsupervised clustering analysis was applied to identify different pyroptosis regulation patterns. By using the consensus clustering algorithm, the cluster stability and the number of clusters were obtained. To identify and ensure the stability of our classification, we utilized the “ConsensuClusterPlus” R package [25] and performed 1000 times repetitions.

### Annotations of gene sets and gene set variation analysis (GSVA)

In order to study the differences between the mechanisms of pyroptosis in biological processes, we performed GSVA enrichment analysis utilizing the “GSVA” R package [26]. The gene sets of “c2.cp.kegg.v7.4.-symbols” were downloaded from the MSigDB database for running GSVA analysis. A cutoff value of FDR < 0.05 was used to assess the significance level in the “clusterProfiler” R package. The pyroptosis-related signaling pathways were statistically significant if the cutoff value was less than 0.05.

### Single sample gene set enrichment analysis (ssGSEA) algorithm estimate cell infiltration in the TME

A ssGSEA algorithm was used to quantify the relative abundance of 23 types of immune cells in the TME. The genome used to label each type of TME infiltrating immune cells was obtained from the study of Charoentong, which stores multiple subtypes of immune cells, involving regulatory T cells, macrophages, natural killer T cells, activated CD8+ T cells, etc. (Table S4) [27, 28].

### Differentially expression genes (DEGs) in distinct pyroptosis regulation patterns

We identified two distinct pyroptosis regulation patterns by analyzing the expression of 33 genes related to pyroptosis. The R package “limma” [29] was used to identify DEGs in pyroptosis samples between different modification clusters. All DEGs were collected in TCGA and CGGA cohorts. DEGs were determined by adjusting the log2 fold change > 1 and *P*-value < 0.01 to determine significance.

### Generation of PSscore

To quantify the differences in pyroptosis regulation patterns among individuals with gliomas, we constructed a scoring system for evaluation, named PSscore, and the procedures were as follows:

We first normalized the DEGs identified from different clusters of pyroptosis samples among all TCGA and CGGA samples. To investigate further, we divided patients into several groups and used the unsupervised clustering method to analyze DEGs. The three gene clusters were defined using the consensus clustering algorithm. Specifically, through a univariate Cox regression model, overlapping genes identified from different pyroptosis gene clusters were selected and used for the OS analysis of each gene. Then, a pyroptosis scoring scheme was developed to quantify the pyroptosis modification level of the patients by using principal component analysis (PCA). Component 1 and component 2 were chosen as signature scores. This method mainly focused the score on the set with the largest block of well correlated (or reverse correlation) genes in this set, while genes that did not track in other sets. PSscore was defined by using a method was analogous to GGI [30, 31]:

1 $${\text{PSscore}} = 0 - \sum {({\text{PC}}1{\text{i}} + {\text{PC}}2{\text{i}})} ,$$

where “i” was the expression of pyroptosis-related genes.

### Correlation of PSscore to other biological processes

According to Mariathasan et al., an analysis of the well-known tumor-associated signatures, this study constructed a correlation between gene sets and some biological processes, including (1) mismatch repair; (2) nucleotide excision repair; (3) WNT targets; (4) epithelial–mesenchymal transition (EMT); (5) angiogenesis signature; (6) DNA damage repair; (7) antigen processing machinery; (8) immune-checkpoint; (9) DNA replication; (10) CD8 T-effector [32–34].

### Correlation analysis between PSscore and TMB

By R package “maftools”, the TCGA mutation data was collected for mutation profile analysis [35], followed by calculation of TMB and correlation analysis between PSscore and TMB. Similarly, the “maftools” R package also extracted mutational signatures from TCGA genome data.

### The immunophenoscore (IPS) analysis quantify the immunotherapy response

In order to predict immune checkpoint inhibitors (ICIs) therapy better, the correlation between PSscore and expression of ICI genes was investigated, and followed analysis was by the acquisition of IPS from The Cancer Immunome Atlas (TCIA) (https://tcia.at/home) [27].

### Clinical glioma specimens for our independently external validation cohort

Between July 1, 2017, and December 30, 2019, ten human glioma tissues specimens were obtained from the Affiliated Hospital of Qingdao University, and all specimens were pathologically confirmed. The study was approved by the ethical review committee of the Affiliated Hospital of Qingdao University (No: QYFYWZLL26371). The clinicopathological features were listed in Table S5.

### Detected the expression of pyroptosis-related DEGs in clinical samples

RT2 Profiler™ PCR Arrays (Qiagen) were used to profile the expression of 1208 pyroptosis-related DEGs per sample. The patient-derived fresh specimens were directly extracted total RNA and synthesized cDNA, then the expression of DEGs was quantified using RT^2^ Profiler™ PCR Arrays method by the Berry Genomics Company (China). Relative gene expression was calculated by the 2−ΔΔ Ct method and was normalized to HPRT gene expression. To calculate the PSscore, we corrected the possible batch effects by the ComBat function from the “sva” R package on our independently external validation cohort and TCGA-CGGA meta-cohort.

### Immunohistochemistry

Immunohistochemical staining of paraffin sections of human glioma specimens was performed using antibodies to GSDMD. Mouse monoclonal anti-GSDMD antibody (BOSTER; M02842-1; 1:100 dilution), coupled with biotinylated anti-goat IgG secondary antibody (1:200 dilution). Sections were washed in PBS after antibody staining, biotin-streptavidin and colorimetric reagents were added for signal amplification. Subsequently, DAB and hematoxylin stains were used to visualize, and hematoxylin stain was used in the nucleus.

### Immunofluorescence

For colocalization analysis PD-1 and CD3, frozen sections were incubated with mouse anti–human PD-1 antibody (Invitrogen; REF: 14-9969-80; 1:100 dilution) and rabbit-anti-human CD3 antibody (Invitrogen; REF: 14-0032-81; 1:100 dilution;) overnight at 4 °C. Sections were counterstained with Goat Anti-mouse IgG H&L/Cy3 (Bioss; 1:500 dilution) and Donkey Anti-rabbit IgG H&L/FITC (Bioss; 1:500 dilution).

### Statistical analysis

In this study, we used spearman and distance correlation analysis to calculate the correlation coefficient between the expression of pyroptosis-related genes and infiltrative immune cells in TME. Compare the differences among three or more groups by one-way ANOVA and Kruskal–Wallis test [36]. The “survminer” R package was used to determine each dataset subgroup’s cutoff point based on a correlation between the PSscore and patients’ survival. The “surv-cutpoint” function was used to find the maximum rank statistic for dichotomizing PSscore. And then, through the maximally selected rank statistics, we divided patients into low and high PSscore groups. We determined the most optimal cutoff for continuous variables based on log-rank statistics. In the OS analysis, survival curves were derived from Kaplan–Meier methods, and the significance of differences was assessed using log-rank tests. We calculated hazard ratios (HR) using a one-variate Cox regression model for pyroptosis-related genes.

Moreover, we ascertained the independent prognostic factors through a multivariable Cox regression model. Final multivariate prognostic analysis was carried out on patients with detailed clinical data. The results of multivariate OS analysis for PSscore were visualization by the “forestplot” R package in the TCGA-CGGA meta-cohort. Using the “pROC” package in R, we calculated the area under the curve (AUC) and assessed the specificity and sensitivity of the PSscore using a receiver operating characteristic (ROC) curve. The TCGA cohort was analyzed using the waterfall function of the “maftools” R package to determine the mutation landscape of patients with high and low PSscore subtypes. “RCircos” R package was used to plot the chromosomal copy number variation landscape [35]. All statistical *p* values were on two sides, *p* < 0.05, with statistical significance. In R 4.1.0, all data processing was completed.

## Results

### The landscapes of genetic variation and expression difference in pyroptosis-related genes

**Fig. 1:**
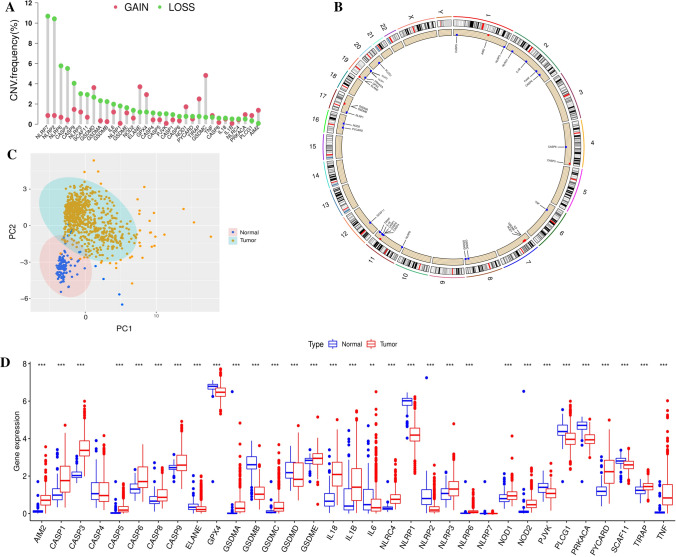
Genomic and expression variation landscape of pyroptosis-related genes in gliomas. **a** The CNV mutation frequency of 33 pyroptosis-related genes was widespread. This column represented the variation frequency. Green dots represented deletions; red dots represented amplifications. **b** The location of 33 pyroptosis-related genes on chromosomes. **c** Principal component analysis (PCA) was performed on 33 pyroptosis-related genes to distinguish normal from the tumor; normal, blue; tumor, yellow. The expression profiles of 33 pyroptosis-related genes to distinguish tumors from normal samples in TCGA and GTEx cohorts. According to the 33 pyroptosis-related genes expression profiles, identifying two subgroups without intersection indicated distinct tumors and normal samples. **d** The different expression levels of 33 pyroptosis-related genes between normal and glioma samples. The statistical *p*-value was represented by asterisks (*p < 0.05; **p < 0.01; ***p < 0.001)

The roles of 33 pyroptosis-related genes in glioma were studied. Figure 1a summarized the gene-related process of pyroptosis and potential biological functions. For the first time, the relative conservation in the somatic mutation of 33 pyroptosis-related genes was determined in glioma. Seventy-seven experienced genetic alterations in pyroptosis-related genes among 896 TCGA glioma samples, with only a frequency of 8.59%. The result indicated that the frequency of pyroptosis-related genes mutations was not high in the glioma. It was found that the NLRP2, NLRP3, NLRP7, PLCG1, SCAF11, NLRP1, NOD1, CASP1, and NOD2 exhibited relatively high mutation frequency (1%), while CASP3, GSDME, IL1B, PJVK, TIRAP did not show any mutations in glioma samples (Fig. S1b). Additionally, co-occurrences of mutations were found significant between NOD1 and SCAF11, NOD2 and NLRP7, NLRP1 and PLCG1 (Fig. S1c). Further analysis of 33 pyroptosis-related genes showed that CNV mutations were commonly observed. The investigation of CNV mutations frequency revealed that CNV mutations exhibited pervasive variation in 33 pyroptosis-related genes. CASP1/3/4/5/6/9, NLRP2/3/6/7, IL6, IL18, GSDME, GSDMA, GSDMB, NLRC4, NOD2, and PYCARD showed widespread CNV deletions. In contrast, GSDMC, ELANE, GSDMD, GPX4, TIRAP, NOD1, AIM2, PRKACA, and PLCG1 had relatively high copy number of CNV amplification (Fig. 1a). Figure 1b showed the locations of CNV alterations of 33 pyroptosis-related genes on chromosomes. Based on the expression of these 33 pyroptosis-related genes and the PCA method, we could clearly distinguish glioma samples (TCGA and CGGA meta-cohort) from normal samples (TCGA and GTEx meta-cohort) (Fig. 1c). The analysis demonstrated that in comparison with normal brain tissues, 20 pyroptosis-related genes were upregulated in the tumor (e.g. CASP1 and CASP3), and 11 pyroptosis-related genes were significantly downregulated in the tumor (e.g. GSDMB and NLRP1) (Fig. 1d).

### Pyroptosis regulation patterns as cluster A–B predicted prognostic value

**Fig. 2:**
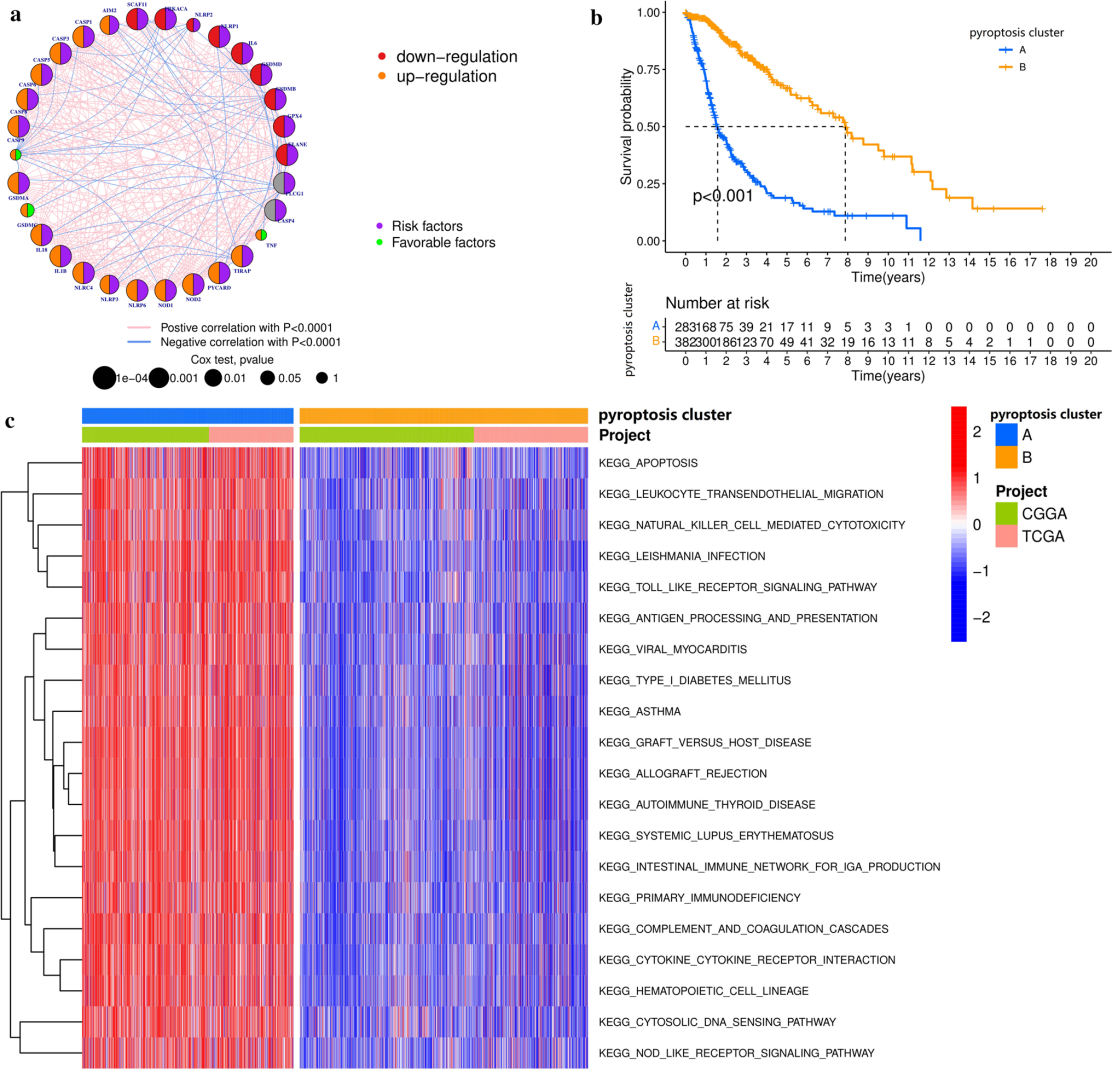
Prognostic and biological characteristics of pyroptosis modification patterns. **a** The interaction between pyroptosis-related genes in glioma. The size of the circles represented the effect of each gene on prognosis, and the log-rank test range of values was *p* < 0.0001, *p* < 0.001, *p* < 0.01, *p* < 0.05, and *p* < 0.1. The lines linking related genes showed their interactions. Negative correlation, blue; Positive correlation, pink. Risk factors of prognosis were marked with purple; Favorable factors of prognosis were marked with green. The gene expression was upregulated or downregulated in the tumor marked orange or red, respectively. **b** The survival curves of the pyroptosis clusters were estimated by the Kaplan–Meier plotter (*p* < 0.001, log-rank test). **c** Gene set variation analysis (GSVA) enrichment analysis showed distinct pyroptosis regulation patterns with different biological pathways activated. Visualizing biological processes by heatmap, activated pathways, red; inhibited pathways, blue

We enrolled two CGGA cohorts and one TCGA cohort with OS and clinical data to form one TCGA-CGGA meta-cohort (mRNAseq-693, mRNAseq-325, and TCGA-LGGGBM cohorts; Table S2). The prognostic value of pyroptosis-related genes was shown in a univariate Cox regression model for glioma patients (Fig. S1d). To overviewed the protein-protein interactions and molecular regulations, we constructed the pyroptosis-regulated genes network, the comprehensive landscape of pyroptosis gene interactions, gene connections, and prognostic significance for glioma patients visualized (Fig. 2a and Table S6). In addition, we found that the expression levels of the pyroptosis-related genes seemed to be remarkably correlated with each other and the different categories. The above results demonstrated that the genes involved in pyroptosis might be fundamental to determining pyroptosis-related modification patterns. The “ConsensusClusterPlus” R package was used to classify patients with qualitatively different pyroptosis regulation patterns according to the expression of these pyroptosis-related genes. The two distinct modification patterns were eventually identified using unsupervised clustering, including 715 cases in pattern A and 975 cases in pattern B. We termed these patterns as pyroptosis cluster A–B (Fig. S2a and Table S7). The expression heatmap in Fig. S2a showed that most pyroptosis-related genes were upregulated in the pyroptosis cluster-A. In contrast, the pyroptosis cluster-B modification pattern showed a significant survival advantage over cluster-A (Fig. 2b). Unlike most other tumors, these results suggested that pyroptosis played a negative role in the prognosis of glioma.

### Different pyroptosis regulation patterns showed distinctive TME cell infiltration characteristics

**Fig. 3:**
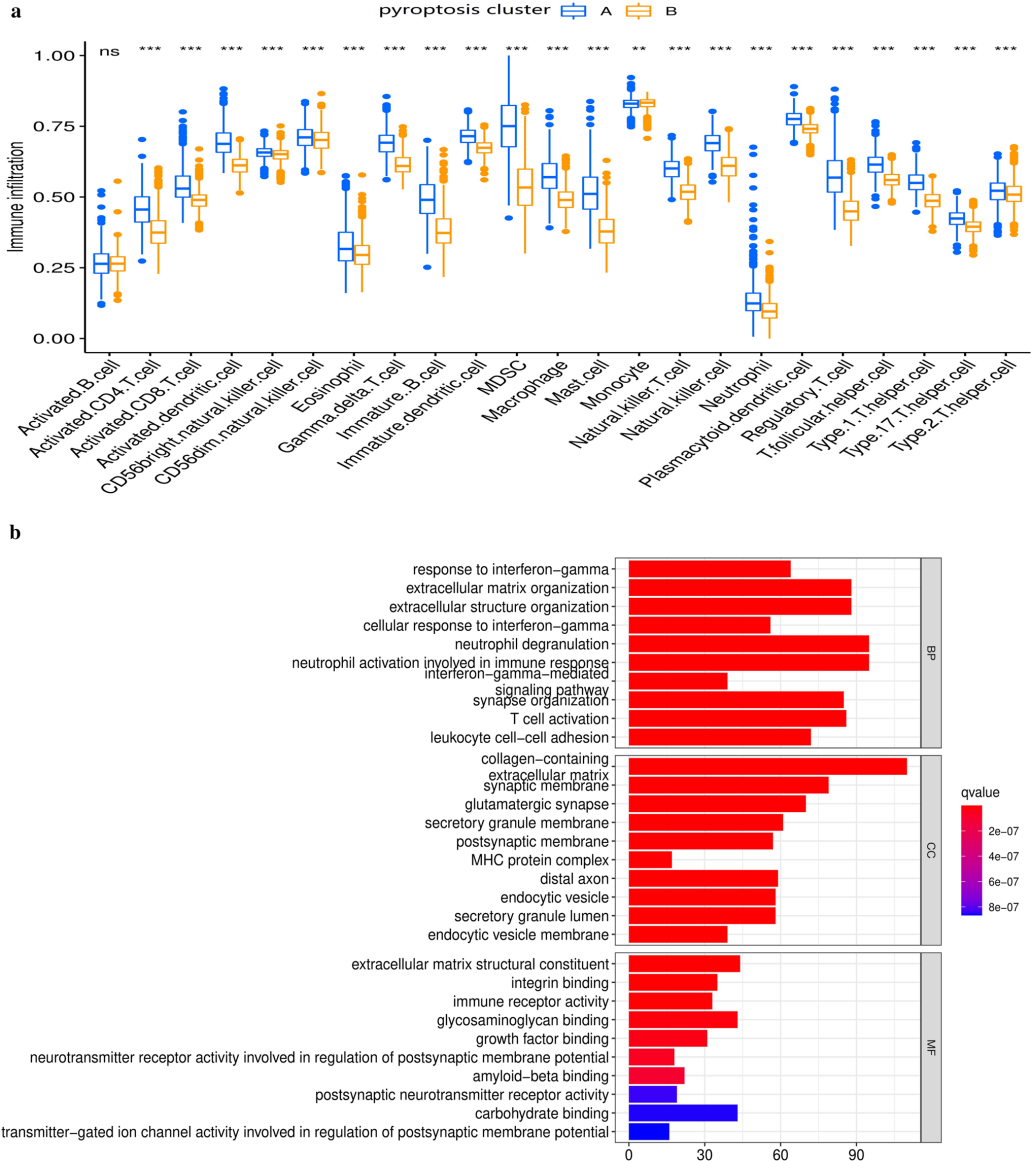
The characteristics of the tumor microenvironment (TME) cell infiltration and transcriptome in distinct pyroptosis regulation patterns. **a** The composition of TME infiltrating cells in two pyroptosis regulation patterns. c (*p < 0.05; **p < 0.01; ***p < 0.001). **b** Functional annotation for differentially expressed genes (DEGs) of pyroptosis regulation patterns using GO enrichment analysis. The number of genes enriched was represented by the length and depth of bar plots

By analyzing GSVA enrichment data, we explored the biological behavior of the two different pyroptosis regulation patterns. As shown in Fig. 2c and Table S8, pyroptosis cluster-A was significantly enriched in innate immune response pathways, such as NOD-like receptor signaling pathway, apoptosis signaling pathway, etc., while pyroptosis cluster-B presented an immunosuppressive enrichment state. To our interest, the TME cell infiltration characteristics of these two pyroptosis regulation patterns were also significantly different. Following analysis of infiltrative cells in the TME, we found that pyroptosis cluster-A was rich in immune cell infiltration (Fig. 3a and Table S7). In cluster-A, which showed an immune-inflamed phenotype, there was a high level of infiltration and immune activation; in cluster-B, there was a low level of infiltration and a low level of immune reactivity (Figs. 2c and 3a). The GSVA found that the pyroptosis clusters significantly affected immune activation and infiltration, suggesting cluster-A associated with innate immune activation was much more significant than adaptive immune responses and led to a poor prognosis (Fig. 2b).

Then spearman’s correlation analyses were used to examine the relationship between TME infiltration cell and pyroptosis-related genes (Fig. S3a). We were concerned about the GSDMD gene, a crucial effector in pyroptosis, and it showed a significant positive correlation with abundant TME-infiltrating cells. We also found that pyroptosis cluster-A showed a significantly higher GSDMD expression than pyroptosis cluster-B (Fig. S3b). We quantified immune cells infiltrate between the high and low expression levels of GSDMD using the ESTIMATE algorithm. The results revealed that the TME with high expression of GSDMD exhibited a high immune score and showed a significant increase in immune cell infiltration (Fig. S3c, d). This result was consistent with the result in Figs. 2c and 3a. After that, we assessed the specific difference between patients who expressed high or low GSDMD about 23 kinds of TME infiltrating cells. A significant increase in TME immune cells was detected in GSDMD high-expressed tumor, compared to GSDMD low-expressed tumors (Fig. S3d). As a consequence of the elevated expression of GSDMD, we observed an increase in the expression of immune, such as MHC, adhesive molecules (Fig. S3e). Tumors expressing high levels of GSDMD displayed a noticeable increase in immune activation pathways during pathway enrichment analyses, such as T cell receptor, Toll-like receptor, etc. (Fig. S3f). In addition, it was discovered that the enhancements in immune-related pathways were accompanied by an increased expression of the immunologic checkpoint molecules such as PD-1 (PDCD1) (Fig. S3e, f). We could infer from the above results that pyroptosis led to activation of innate and adaptive immune responses and increased immune cell infiltration. However, this phenomenon did not provide an increased benefit in the prognosis of glioma. This finding could be due to pyroptosis in glioma caused uncontrolled innate immune responses, and excessive inflammatory mediators would cause organ failure.

### Generation of pyroptosis gene signatures and functional annotation

**Fig. 4:**
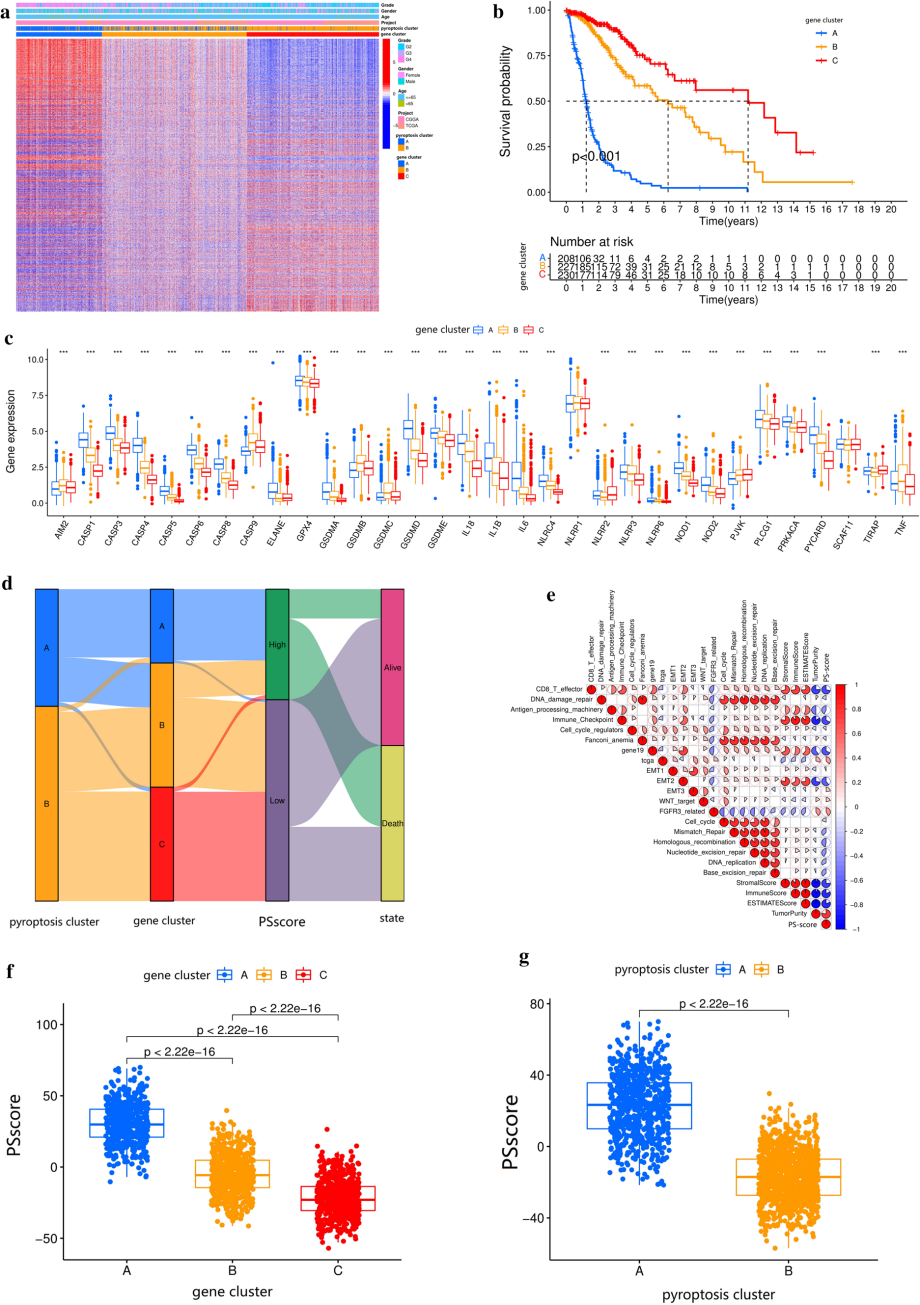
The DEGs of pyroptosis regulation patterns were exploited to generate pyroptosis signatures. **a** The DEGs of pyroptosis regulation patterns to classify patients into three different genomic subtypes by unsupervised clustering, termed as pyroptosis gene cluster-A/B/C, respectively. The gene clusters, pyroptosis clusters, tumor grade, gender, and age were used as patient annotations. **b** The survival curves of the pyroptosis-related genes signatures were estimated by the Kaplan–Meier plotter (*p* < 0.001, log-rank test). **c** The 33 pyroptosis-related genes expression difference in these three gene clusters. The upper and lower edge of each box represented upper and lower quartiles. Lines represented medians, and dots indicated outliers. Asterisks represented the statistical p-value (*p < 0.05; **p < 0.01; ***p < 0.001, one-way ANOVA test). **d** The alluvial diagram demonstrated the changes of pyroptosis clusters, gene clusters, PSscore, and survival status (state). **e** Correlations among PSscore, the different gene signatures and TME, TCGA and CGGA glioma cohort using Spearman analysis. Negative correlation, blue; positive correlation, red. **f** The differences in PSscore of the three gene clusters in the TCGA and CGGA cohorts (*p* < 0.001, Kruskal–Wallis test). **g** The differences in PSscore of the two pyroptosis clusters in the TCGA and CGGA cohorts (*p* < 0.001, Kruskal–Wallis test)

We investigated pyroptosis-related DEGs from the TCGA-CGGA meta-cohort to further understand the biological behavior of pyroptotic clusters (Table S9). GO enrichment analysis of DEGs was conducted using the “clusterProfiler” R package. The biological processes that exhibited significant enrichment were summarized (Table S10). Notably, the DEGs showed a remarkable enrichment in pathways related to immunity and anabolism (such as T cell activation, immune receptor activity, glycosaminoglycan binding, carbohydrate binding), which suggested the pyroptosis played a non-negligible role in the regulation of immune responses in TME (Fig. 3b). As a next step for confirming this regulation mechanism, unsupervised clustering analyses were then carried out based on the DEGs to sort patients into different genomic subtypes. The result was similar to pyroptosis regulation pattern clustering in Fig. 2c. The unsupervised clustering algorithm distinguished three distinct genomic phenotypes of pyroptosis modification, which we called pyroptosis gene clusters A/B/C, respectively (Fig. 4a and Fig. S4a–d and Table S11). An analysis revealed that different signature genes were associated with three distinct gene-clusters (Fig. 4a). Most of the glioma tumors in the pyroptosis gene cluster-A pattern also characterized high differentiation grades (G4) and age ≥ 65 years. Both clusters B and C of pyroptosis-related DEGs showed opposite patterns (Fig. 4a). The gene cluster-A was correlated to a worse OS in 504 patients with glioma (Fig. 4b). In contrast, an excellent OS was observed in the gene cluster-C (558 patients). The 628 patients in the gene cluster-B had an intermediate OS (Fig. 4b). The noticeable difference in the expression of pyroptosis-related genes was observed in all three pyroptosis gene-clusters, which was generated based on the pyroptosis regulation pattern (Fig. 4c).

We found that the gene cluster-A was relatively high in the expression of transcripts associated with immune activation and immune checkpoints. It was suggested that the gene cluster-A was an immune-upregulated group (Fig. S4e). While the gene cluster-C showed immunosuppression and a low degree of inflammation was significantly associated with a low degree of immune response and infiltration (Fig. S4f). Furthermore, it was found that almost all patients in the gene cluster-A were correlated with high PSscore and poorer OS compared to the gene cluster-B or C, as shown in Fig. 4b, d.

As shown above, pyroptosis regulation played an influential regulatory role in determining the composition and function of TME landscapes. Nonetheless, the analyses were based on population, not accurately predicting the types of pyroptosis for individual patients. Based on these related genes, pyroptosis regulation exhibited individual heterogeneity and complexity. We established a scoring system to quantify pyroptosis modification patterns with glioma. And we termed it as PSscore. Individual patients’ attributed changes were visualized with the alluvial diagram (Fig. 4d). The correlation between the well-known tumor-associated signatures and the PSscore was also examined to illustrate the pyroptosis signature characteristics (Fig. 4e and Table S12). The Kruskal–Wall test identified pyroptosis gene clusters that demonstrated significantly different PSscore levels. Gene cluster-A had the highest PSscore, while gene cluster-C had the lowest PSscore, suggesting that a low PSscore might be associated with immunosuppression signatures, whereas a high PSscore might indicate immune activation (Fig. 4f). In addition, pyroptosis cluster-A showed a significantly higher PSscore than pyroptosis cluster-B (Fig. 4g). These results indicated that the PSscore could excellently quantify the level of pyroptosis in glioma and reflected the pyroptosis regulation patterns.

### Characteristics of pyroptosis regulation patterns in different TCGA glioma somatic mutation subtypes

**Fig. 5:**
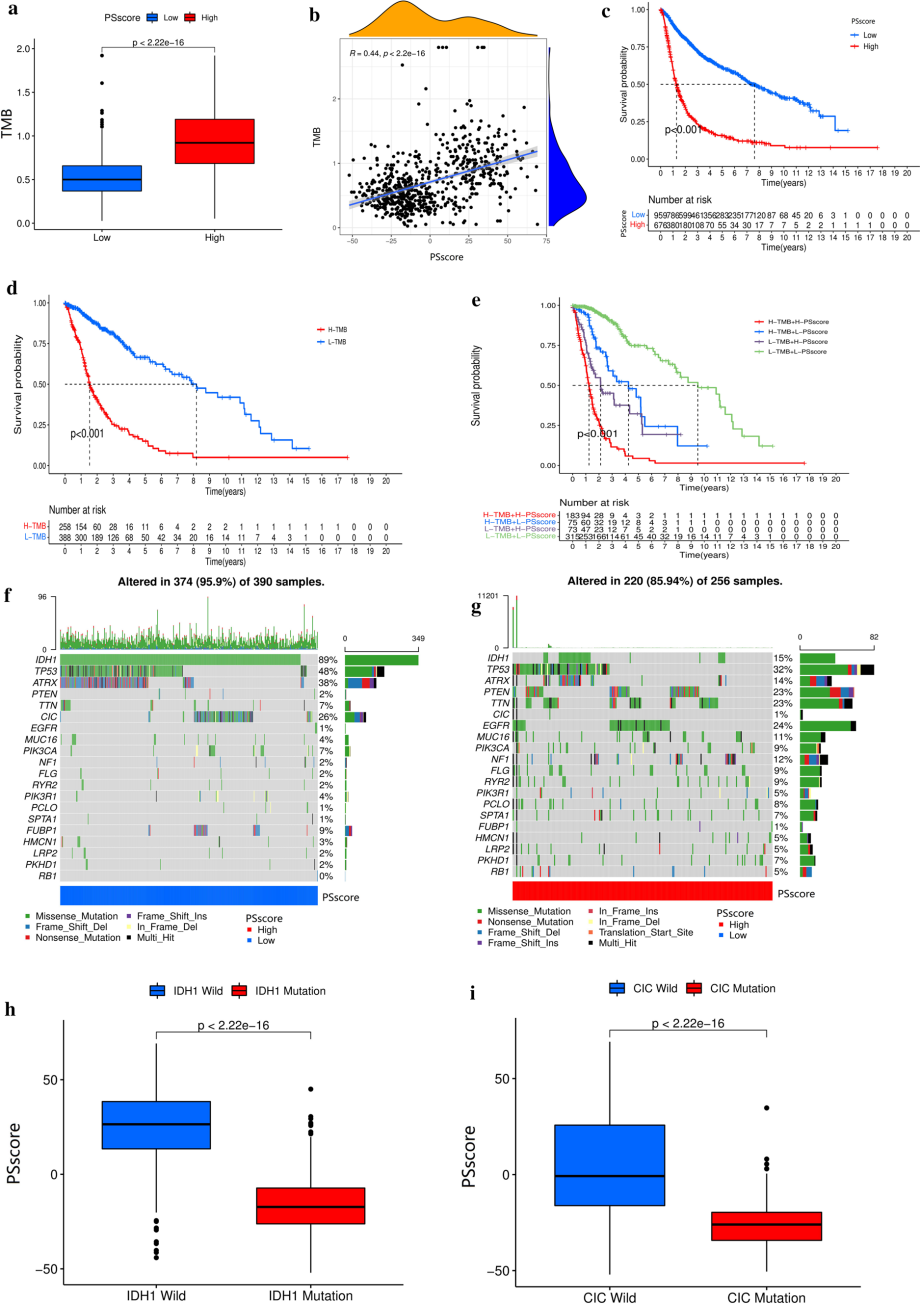
Characteristics of pyroptosis regulation patterns in different TCGA tumor somatic mutation subtypes. **a** TMB differences between low PSscore and high PSscore groups [*p* < 0.001, Wilcoxon test]. **b** A significant positive correlation between the PSscore and TMB (*p* < 0.001). **c** The survival analysis with Kaplan–Meier curves of the low and high PSscore (*p* < 0.001, log-rank test). **d** The survival analysis with Kaplan–Meier curves of the low and high TMB groups (*p* < 0.001, log-rank test). H, high; L, Low; TMB, Tumor Mutation Burden (*p* < 0.001, log-rank test). **e** Survival analysis with Kaplan–Meier curves for low and high PSscore with TMB patients (*p* < 0.001, log-rank test). **f**, **g** The waterfall chart of gene mutation status was generated by those with low PSscore (**f**) and high PSscore (**g**). **h**, **i** The PSscore’s difference between IDH1 wild group and IDH1 mutant group (**h**), CIC wild group and CIC mutant group (**i**). The upper and lower edge of each box represented upper and lower quartiles. Lines represented medians, and dots indicated outliers (*p* < 0.001)

The multivariate analysis for TCGA-CGGA meta-cohort studies confirmed PSscore as a potential independent prognostic biomarker in glioma patients (Fig. S5a). Moreover, there were significant differences in gene mutation frequencies between high PSscore and low PSscore groups, such as IDH1, PTEN, CIC, etc. Quantification analyses of TMB also confirmed a positive association between higher PSscore tumors and higher TMB (Fig. 5a). Moreover, there was a positive correlation between PSscore and TMB (Fig. 5b).

In addition, accumulated evidence showed that patients with high TMB experienced a perseverative response to anti-PD-1/PD-L1 or CTLA-4 immunotherapy [37]. It suggested a potential predictive value of the PSscore in immunotherapy. Thus, we postulated that different tumor pyroptosis regulation patterns might be crucial in determining immunotherapy response against PD-1/PD-L1 or CTLA-4. Taken together, our study suggested that a high PSscore was significantly correlated with high immune responses, whereas a low PSscore was significantly correlated with immune suppression. The PSscore could evaluate the pyroptosis regulation pattern of an individual tumor and its immune cell infiltration, TMB, immune checkpoint therapy outcome. In the next step, we sought to examine the impact of PSscore on patient prognosis. Patients were divided into low or high PSscore groups determined by the “survminer” R package. The low PSscore patients showed an obvious survival advantage (Fig. 5c). Moreover, patients with low TMB also demonstrated significant survival benefits (Fig. 5d). Therefore, the PSscore signature was assumed to predict prognosis in glioma patients with various degrees of TMB. Patients with low PSscore and low TMB showed a significant survival advantage. In contrast, patients with high PSscore and TMB had the worst survival outcome. This result implied that a combination of PSscore and TMB to predict prognosis was better than either factor isolated (Fig. 5e). Then, during the analysis, we compared the somatic mutation distribution between individuals with low- and high-PSscore within the TCGA-glioma cohort using the “maftools” R package. As shown in Fig. 5f, g, patients with glioma had extensive tumor mutations. We focused on the specific altered genes in TCGA, such as IDH1 (The most common mutation in glioma) and CIC (specific-mutation in low PSscore group), which had significantly low PSscore compared to wild type (Fig. 5h, i). The above results indicated that a combination of PSscore and somatic mutation had better clinical application value than applied PSscore alone.

### Verification of the role of pyroptosis regulation patterns in immune checkpoint inhibitors immunotherapy

To test PSscore’s model stability, we applied PSscore signature to a series of different subgroups in TCGA and CGGA cohorts to verify its OS value (Age > 65 subgroup, *p* = 0.002; Age ≤ 65 subgroup, *p* < 0.001; Grade G2–3 subgroup, *p* < 0.001; Grade G4 subgroup, *p* < 0.001; Gender Female subgroup, *p* < 0.001; Gender Male subgroup, *p* < 0.001; Fig. S6a–f). These data indicated that low PSscore correlated with better clinical outcomes in different types of glioma patients. The predictive advantage evaluated with 1-, 3- and 5-year ROC curves was reflected in TCGA and CGGA cohorts (Fig. S6g–i).

**Fig. 6:**
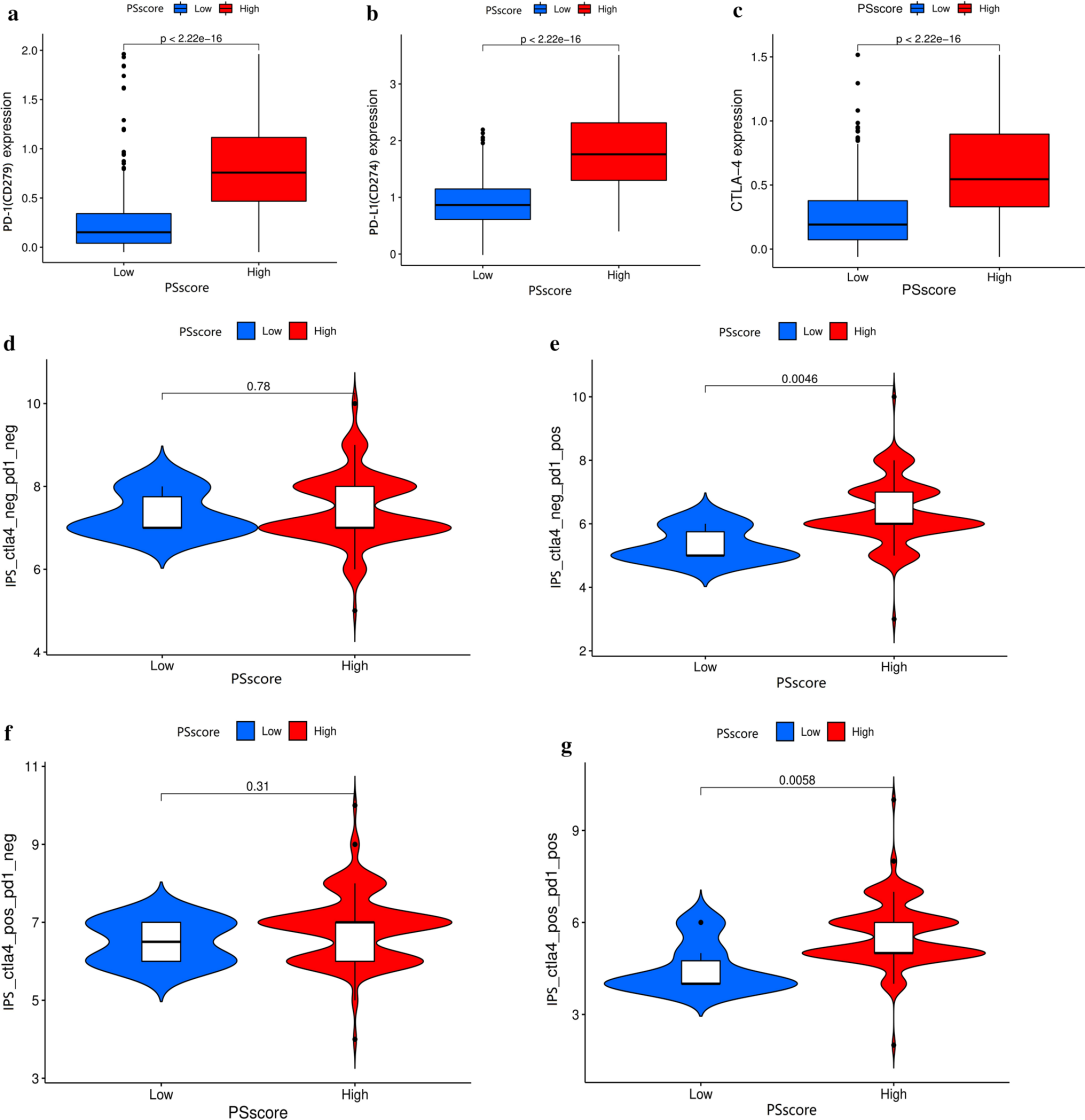
The role of pyroptosis regulation patterns in ICI immunotherapy. **a**–**c** A differential expression of the between low and high PSscore groups (**a** PD-1 (CD279); **b** PD-L1 (CD274); **c** CTLA-4; *p* < 0.0001, Wilcoxon test). **d** The immunophenoscore **(**IPS) difference between low PSscore and high PSscore groups in the anti-CTLA4 and anti-PD1 double no-response subtype cohort. **e** The immunophenoscore **(**IPS) different between low PSscore and high PSscore groups in the anti-CTLA4 no-response but anti-PD-1 response subtype cohort. **f** The immunophenoscore **(**IPS) difference between low and high PSscore groups in the anti-CTLA4 response and anti-PD1 no-response subtype cohort. **g** The immunophenoscore **(**IPS) difference between low PSscore and high PSscore groups in the anti-CTLA4 and anti-PD1 double response subtype cohort

Blocking CTLA-4 and PD-1 immunotherapies became a crucial part of cancer therapy [42]. For this purpose, PD-1 (CD279)/PD-L1 (CD274) and CTLA-4 expression were assessed. The result demonstrated that these three genes had high expression in the group with high PSscore, indicating a potential response to anti-PD-1/L1 or CTLA-4 immunotherapy in these patients (Fig. 6a–c). Furthermore, the immunophenoscore (IPS) was a superior predictor of anti-CTLA-4 and anti-PD-1 immunotherapy response. The PSscore was investigated to see if it could predict the outcome of immune checkpoint blockade therapy via analyzing and comparing the IPS in different subgroups. The results implied the significant clinical response and therapeutic advantages to anti-PD-1 immunotherapy (high IPS) in glioma patients with high PSscore compared to those with low PSscore (Fig. 6d–g), but the response to anti-CTLA-4 immunotherapy without significant differences between these two groups. The results (Fig. 6) illuminated that PSscore, a potential biomarker, could predict prognosis and assess glioma immune checkpoint therapy clinical response.

### Validation of the PSscore relationship with immune signatures and the prognostic value in our independently collected external validation cohort

**Fig. 7:**
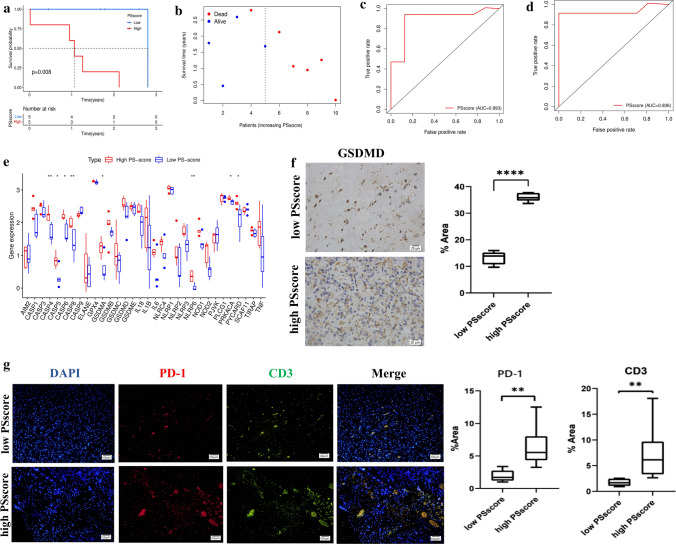
Validation of the PSscore was performed in our independently collected external validation cohort. **a** The survival curves with the Kaplan–Meier curves of the low and high PSscore groups (*p* = 0.008). **b** The distributions of OS status and increasing PSscore in our independently collected external validation cohort. **c**, **d** ROC curve analysis of the predictive value of PSscore in glioma cohorts (**c** 1-year, AUC 0.893; **d** 2-year, AUC 0.936). **e** The 33 pyroptosis related genes expression difference between high and low PSscore in our independently collected external validation cohort (*p < 0.05; **p < 0.01). **f** The expression level of GSDMD upregulated in the high PSscore group compared with the low PSscore group in our independently collected external validation cohort (****p < 0.0001). **g** The expression level of PD-1, CD3 in glioma tissue was upregulated in the high PSscore group compared with the low PSscore group in our independently collected external validation cohort (*p < 0.05; **p < 0.01)

To verify the clinical application value of PSscore for glioma, we adopted a total of 10 glioma clinical specimens were collected from 10 different patients and subjected to analysis for expression of pyroptosis-related DEGs. The genes mRNA levels were measured by qPCR were performed using RT^2^ Profiler PCR Arrays (Qiagen). We further calculated PSscore and divided these patients into two groups with the high and low PSscore by the median of PSscore. We found that the low score group showed an advantage in survival (Fig. 7a). As shown in Fig. 7b, patients with low PSscore had a higher survival probability, while those with high PSscore were closely associated with high mortality risk. The time-dependent ROC curve result showed that during 1 and 2 years, the AUC of prognostic risk assessment models for pyroptosis-related DEGs were 0.893 and 0.936, respectively (Fig. 7c, d). These results were consistent with our previous analysis based on the TCGA -CGGA meta-cohort. It further proved that the PSscore was an excellent prognostic indicator for predicting glioma survival. Moreover, in our clinical specimens, the expression of 33 pyroptosis-related genes was higher in high PSscore tissues than in low PSscore (Fig. 7e). As GSDMD was important in pyroptosis signaling, we subsequently performed immunohistochemistry to analyze the GSDMD expression. The result further validated that GSDMD expression level in high PSscore samples was significantly upregulated compared with low PSscore samples (Fig. 7f). This result was the same as the previous description Fig. S3. To validate the PSscore’s relationship with T cell infiltration and the effects of such interaction in anti-PD-1 immunotherapy, we performed immunofluorescence to analyze CD3 and PD-1 expression in these tissues and found that the CD3+ T cells and CD3+PD-1+ cells significantly increased in the high PSscore group (Fig. 7g). The above results further validated our findings retrieved from the TCGA-CGGA meta-cohort and demonstrated that the PSscore was a valuable marker of glioma prognosis and predictor of ICI immunotherapy.

## Discussion

Increasing evidence indicate that pyroptosis plays an important role in inflammation, immunity, and anti-tumor responses. However, the integrative roles of multiple pyroptosis-related genes are still poorly elucidated. By identifying the role of pyroptosis regulation patterns in TME, we may better understand the anti-tumor immune response and guide the design of more effective immunotherapy [38].

This study found two distinct pyroptosis regulation patterns (pyroptosis cluster A–B) based on 33 pyroptosis-related genes. There was significantly distinct tumor immune cell infiltration characterization in these two patterns. Immune cells were highly infiltrated in cluster-A, while cluster-B had low immune cell infiltration. We focused on GSDMD and analyzed it for further verification because it was well known that GSDMD-mediated pyroptosis regulation could promote immune cell infiltration and immune response in TME. Thus, GSDMD caused a severe intratumoral immune-inflammatory response and negatively impacted glioma patients’ outcomes. By combining TME immune cell infiltrating properties with our immune phenotyping classification, we validated the reliability of our definition and classification of various pyroptosis regulation patterns. Therefore, it was concluded that cluster-A had a more activated innate immunity with a worse prognosis after comprehensively analyzing the TME cell-infiltrating characterization of pyroptosis regulation patterns. Furthermore, many studies also have demonstrated that the upregulated innate immune response could drive poor prognosis in many diseases [39–41].

We further examined the relationships between pyroptosis-related pathways and distinct pyroptosis regulation patterns. A scoring system was established to reckon the pyroptosis regulation pattern for the individual patient with glioma-PSscore. The gene clusters based on pyroptosis signature genes were also associated with immune activation, which was consistent with the clustering of pyroptosis modifications. The study showed that pyroptosis modification was influential in shaping different TME landscapes. Hence, comprehensive assessments of pyroptosis regulation patterns could provide a novel insight into cellular infiltration patterns of TME. Due to individual heterogeneity in pyroptosis modification, evaluation of the pyroptosis modification for each tumor is urgently required. In the pyroptosis regulation patterns, cluster-A had a high PSscore while cluster-B exhibited a lower one. These findings suggested that PSscore contributed to assessing the types of glioma pyroptosis patterns and identifying infiltration patterns of TME. Furthermore, our data also comprehensively analyzed a markedly positive correlation between PSscore and TMB. Finally, the clinical application value of PSscore was further confirmed by investigating our independently collected external validation cohort. The glioma patients with low PSscore accompanied an advanced prognosis. While the glioma patients with high PSscore demonstrated high pyroptosis-related gene expression, higher level of immune cell infiltration, and a poor prognosis. However, reassuringly, our data indicated that the anti-PD-1 immunotherapy responses could be expected in those patients with high PSscore. In summary, our results indicated that pyroptosis activation in glioma might play a significant role in tumorigenesis, progression, and response to immunotherapy.

Developing new approaches to boost anti-tumor immunity is critical because many tumors are non-responsive to immunotherapy due to a lack of tumor-infiltrating immune cells or tumor-released antigens. The emerging view suggests that pyroptosis releases inflammatory mediators and could alter TME through the influx of immune cells and eliciting tumor antigens [42]. This study indicated that the pyroptosis modification pattern was strongly correlated to TME, TMB, and the response to the anti-PD-1/L1 immunotherapy. Moreover, it could be used to establish pyroptosis modification signature to determine outcomes of glioma patients administered anti-PD-1/L1 immunotherapy. This study proved that a high PSscore could form an activated innate immune TME and mediate therapeutic response to PD-1 rather than CTLA-4 immune checkpoint blockade to impact the patients’ particular immunotherapy. Our study demonstrated that pyroptosis regulation patterns played an irreplaceable role in shaping immune TME landscapes, implying that pyroptosis modification could affect immune checkpoint blockade’s efficacy. A more effective immunotherapy prediction strategy could be based on a high PSscore, which indicated high PD-1 expression and high inflammation (high tumor antigen) was beneficial in checkpoint therapy.

In brief, this research provided further guidance for developing effective interventions to improve the prognosis and immunotherapy outcome of patients with glioma. We provided PSscore as a novel indicator, helping for improving the patients’ prognosis and clinical therapeutic efficacy. The PSscore was a comprehensive system to evaluate the pyroptosis regulation patterns, TME cell infiltration, TMB, and response to checkpoint therapy in glioma patients. We also demonstrated that the PSscore could assess patients’ clinicopathological features, including histological tumor grades, molecular subtypes, genetic variation, etc. Additionally, as a biomarker, PSscore might be able to predict survival. More importantly, new insights regarding cancer immunotherapy were gained from this study. All the results indicated that we could reverse the adverse TME cell infiltration characterization to change pyroptosis regulation patterns by regulating pyroptosis phenotype-related genes. Moreover, our study suggested that targeting the pyroptosis signaling pathways was the novel strategy for clinical treatment in glioma. In conclusion, we developed an analysis of pyroptosis regulation patterns and their correlation with glioma. It would benefit the development of target intervention strategies and promote personalized glioma therapy.

## Supplementary Information

Below is the link to the electronic supplementary material.

Supplementary material 1 (PDF 12683.1 kb)

Supplementary material 2 (XLSX 980.6 kb)

## Data Availability

All data from the TCGA, CGGA, GTEx, and outputs from our study are available.
