# Supplementary material for: Comprehensive analysis of pyroptosis regulation patterns and their influence on tumor immune microenvironment and patient prognosis in glioma
Source: Discov Oncol. 2022 Mar 10;13:13. doi: 10.1007/s12672-022-00474-5 (PMC8913830; doi:10.1007/s12672-022-00474-5)
Supplement: Supplementary file 1 — Supplementary material 1 (PDF 12683.1 kb) [file 12672_2022_474_MOESM1_ESM.pdf]

Comprehensive analysis of pyroptosis regulation patterns and their influence on tumor immune microenvironment and patient prognosis in glioma

Discover Oncology ,

Tianyu Fan, Yi Wan, Delei Niu, Bin Wang, Bei Zhang, Zugui Zhang, Yue Zhang, Zheng Gong, Li Zhang;
Corresponding author
Dr. Li Zhang or Dr. Zheng Gong
The Department of Immunology, School of Basic Medicine, Qingdao University 308 Ningxia Road, Qingdao, Shandong Province, China.
E-mail: zhangli_qddx@126.com (Li Zhang) or xblong2000@gmail.com (Zheng Gong)

Fig. S1

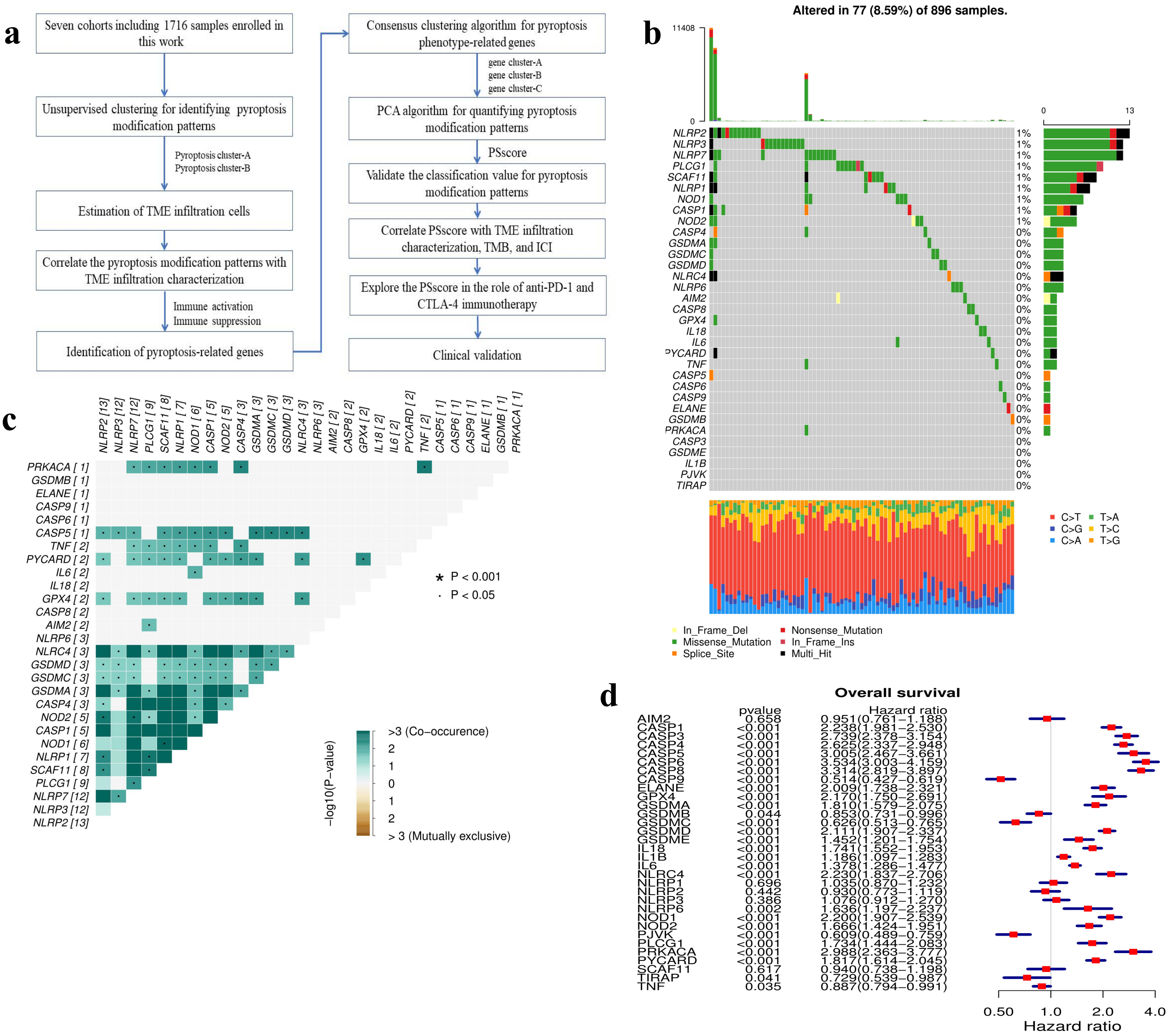

Fig. S1 Study design and overall survival of pyroptosis-related genes a. Overview of this work. **b.** Summarize 896 glioma patients, 77 experienced 33 genetic changes of pyroptosis-related genes, with an incidence of 8.59%. The barplot on the right demonstrated the total number of mutations and the proportion of different mutation types. The upper barplot indicated TMB, and the % number on the right indicated the mutation frequency. The barplot below showed fractions of conversions across samples. Each column represented an individual patient. **c.** The mutual exclusion analyses and mutation co-occurrence for pyroptosis-related genes. Co-occurrence, green; Mutually exclusive, brown. **d.** Univariate Cox regression model predicted the overall survival for pyroptosis-related genes in the glioma cancer cohorts. Hazard ratio <1 and hazard ratio >1 represented protective and risk factors for survival, respectively.

Fig. S2

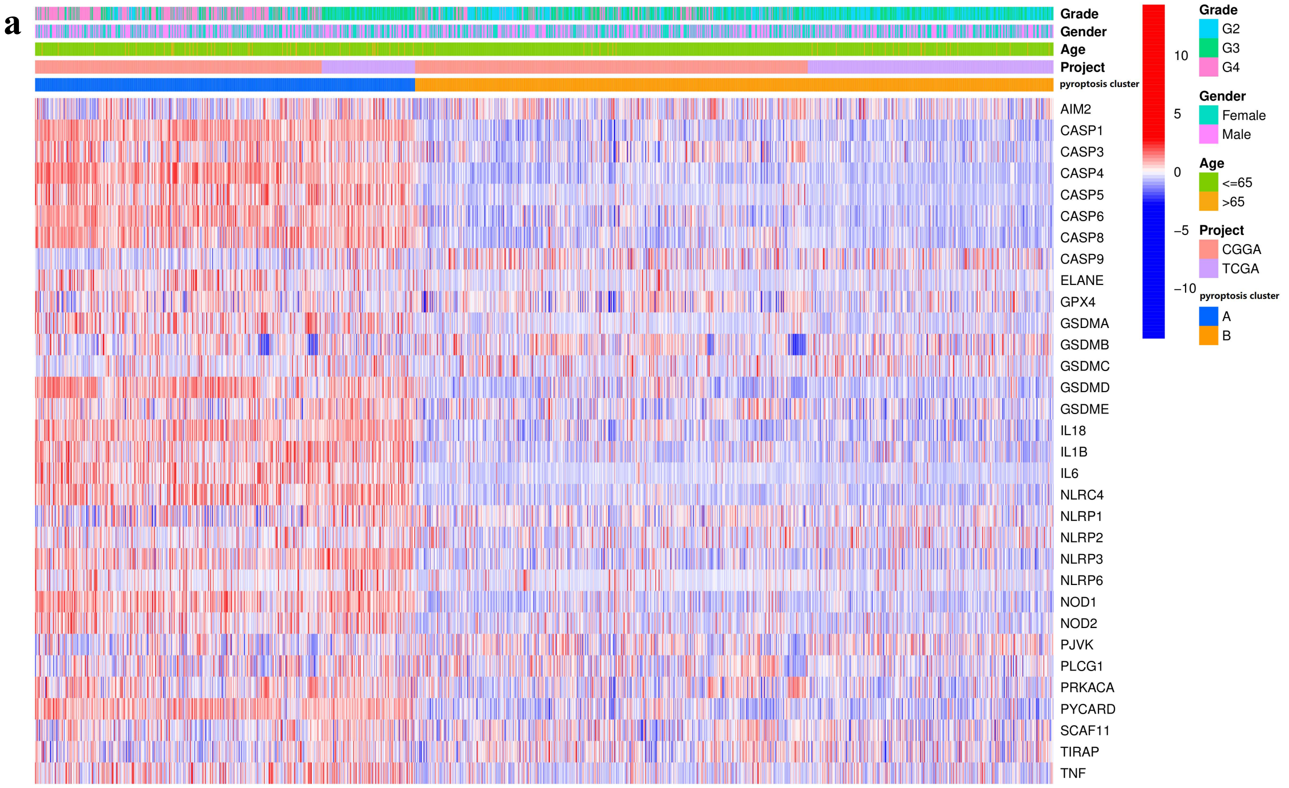

Fig. S2 Correlation between pyroptosis-related genes and characteristics of clinical a. Unsupervised clustering of pyroptosis-related genes in the two independent glioma cohorts. Each column represented patients, and each row represented pyroptosis-related genes.

Fig. S3

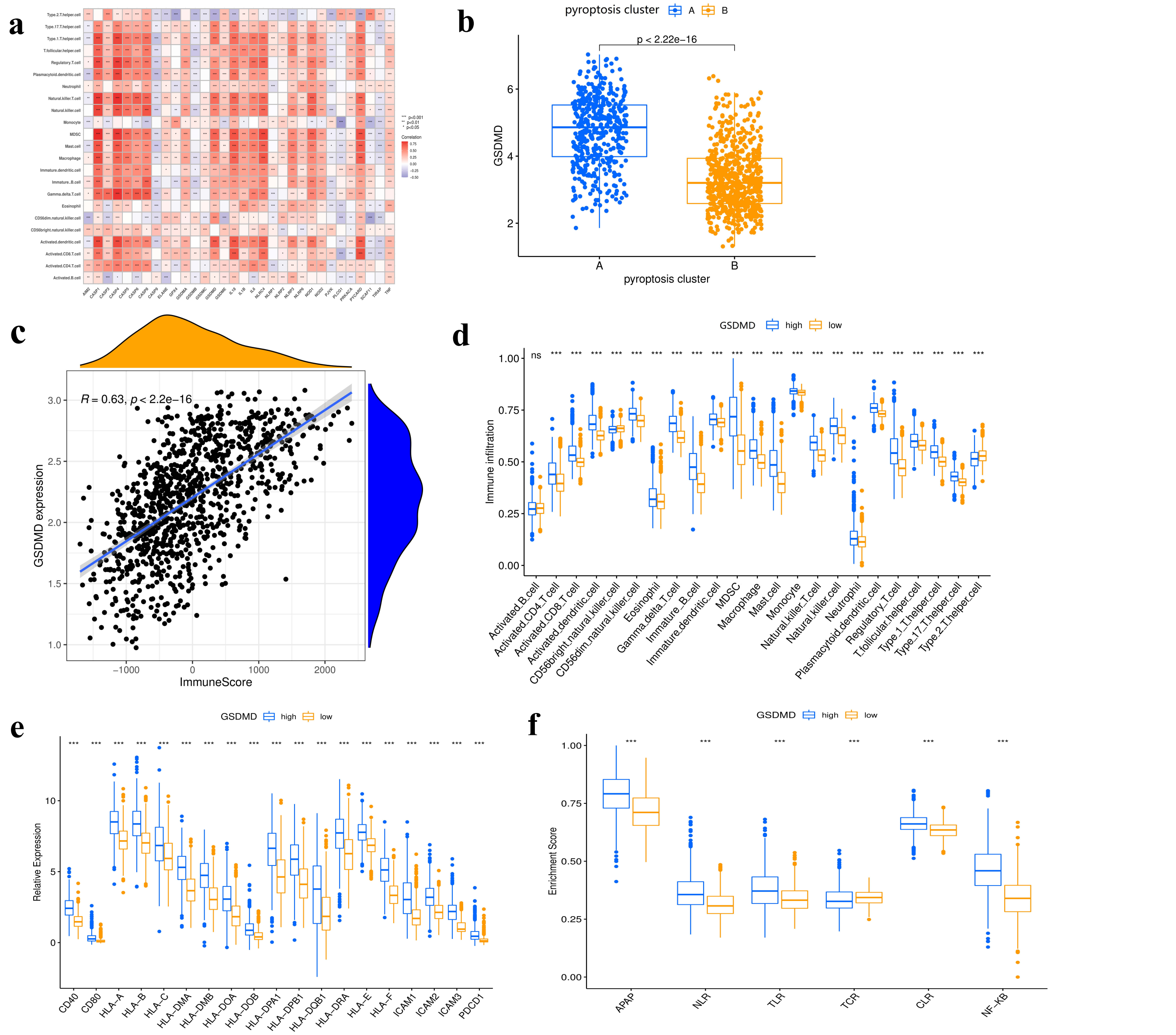

Fig. S3 The roles of GSDMD and the correlation between pyroptosis-related genes and TME infiltration cells a. The correlation between 33 pyroptosis-related gene and different TME infiltration cell by spearman correlation analyses. Positive correlation, red; negative correlation, blue. (*p < 0.05; **p < 0.01; ***p < 0.001). **b.** The differences in GSDMD of the pyroptosis clusters in the TCGA and CGGA cohorts (p< 0.001, Kruskal-Wallis test). **c.** The correlation between immuneScore and GSDMD expression (p<0.0001, R=0.63 Spearman correlation test). **d.** The difference of each TME infiltrating cell between GSDMD low expression and high expression groups (*p < 0.05; **p < 0.01; ***p < 0.001). **e.** The difference in the expression of MHC molecules, costimulatory molecules, and adhesion molecules between GSDMD low expression and high expression groups. Boxes at the upper and lower ends represented the interquartile range. Lines represented medians, and dots indicate outliers. The statistical p-value was represented by asterisks. (*p < 0.05; **p < 0.01; ***p < 0.001). **f.** Differences in immune-activated pathways between GSDMD low and high expression groups. TLR, Toll-like receptor; NOD-like receptor; APAR, antigen processing and presentation; NLR, TCR, T cell receptor; CLR, C-type lectin receptor. (*p < 0.05; **p < 0.01; ***p < 0.001).

Fig. S4

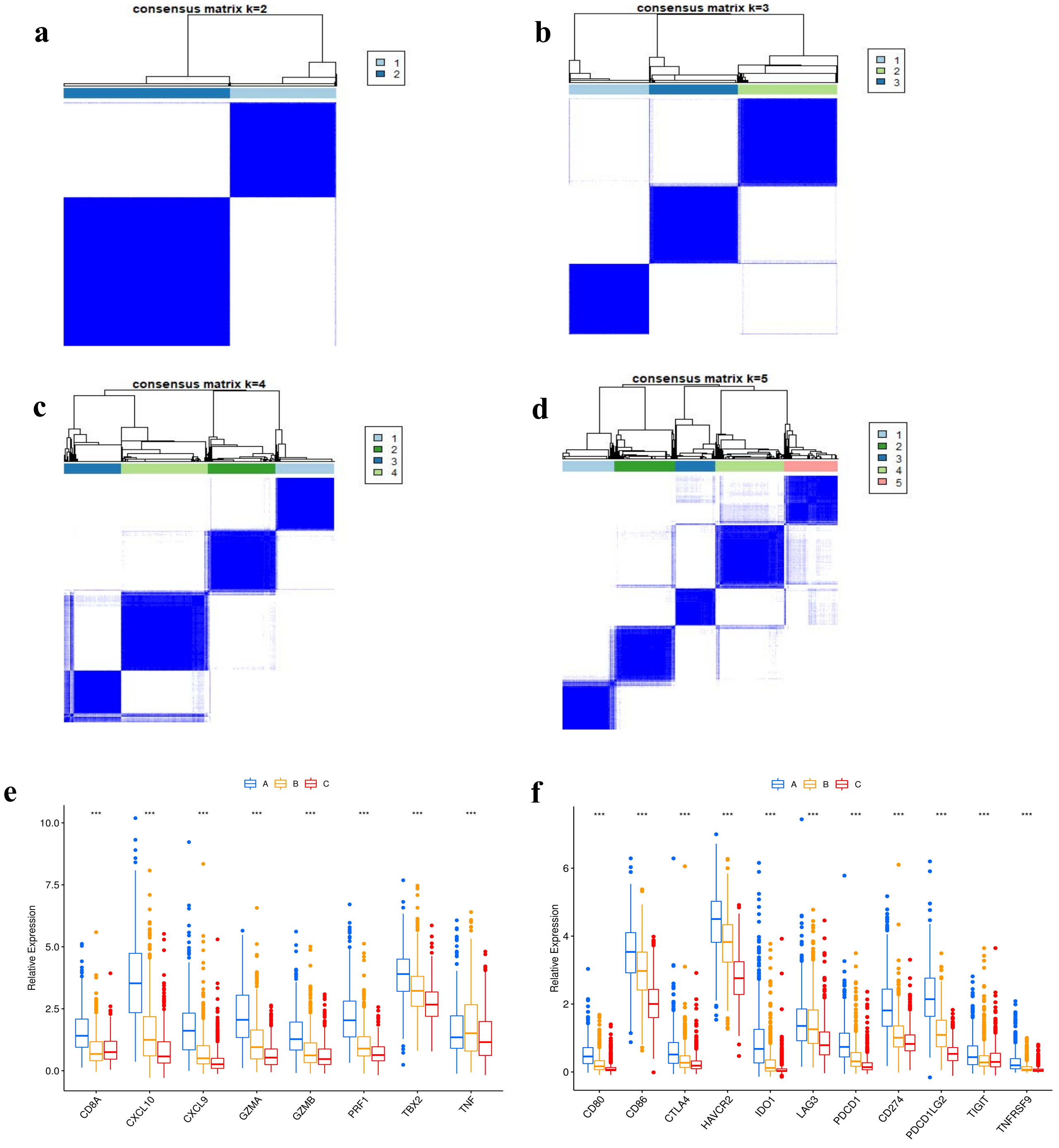

Fig. S4 The gene clusters were generated using unsupervised clustering and the characteristics of known signatures in these distinct gene clusters a-d. Unsupervised clustering of pyroptosis phenotype-related genes and consensus matrices for k=2-5. **e.** Differential expression of immune-activation related genes in three gene clusters. **f.** Differential expression of immune checkpoint related genes in three gene clusters.

Fig. S5

a

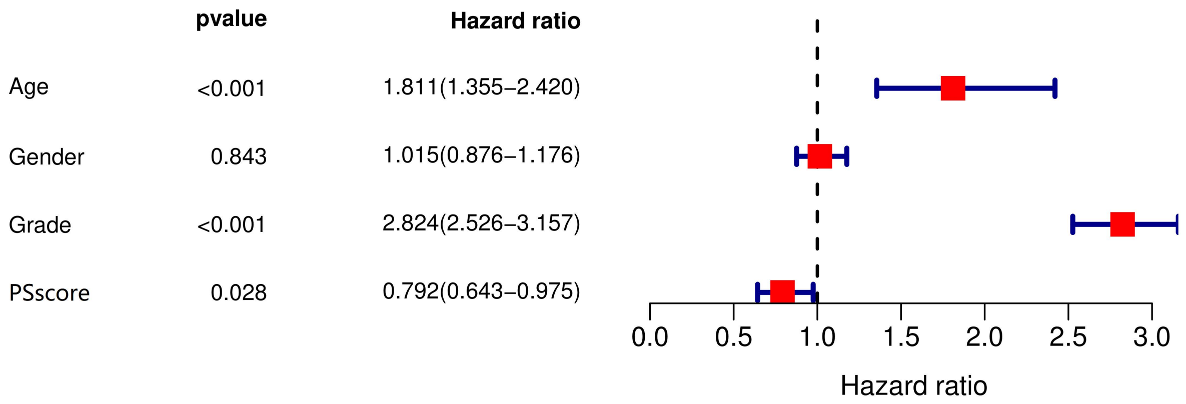

Fig. S5 The correlation between the clinicopathological features and PSscore a. Multivariate Cox regression analysis for PSscore in TCGA and CGGA cohorts.

Fig. S6

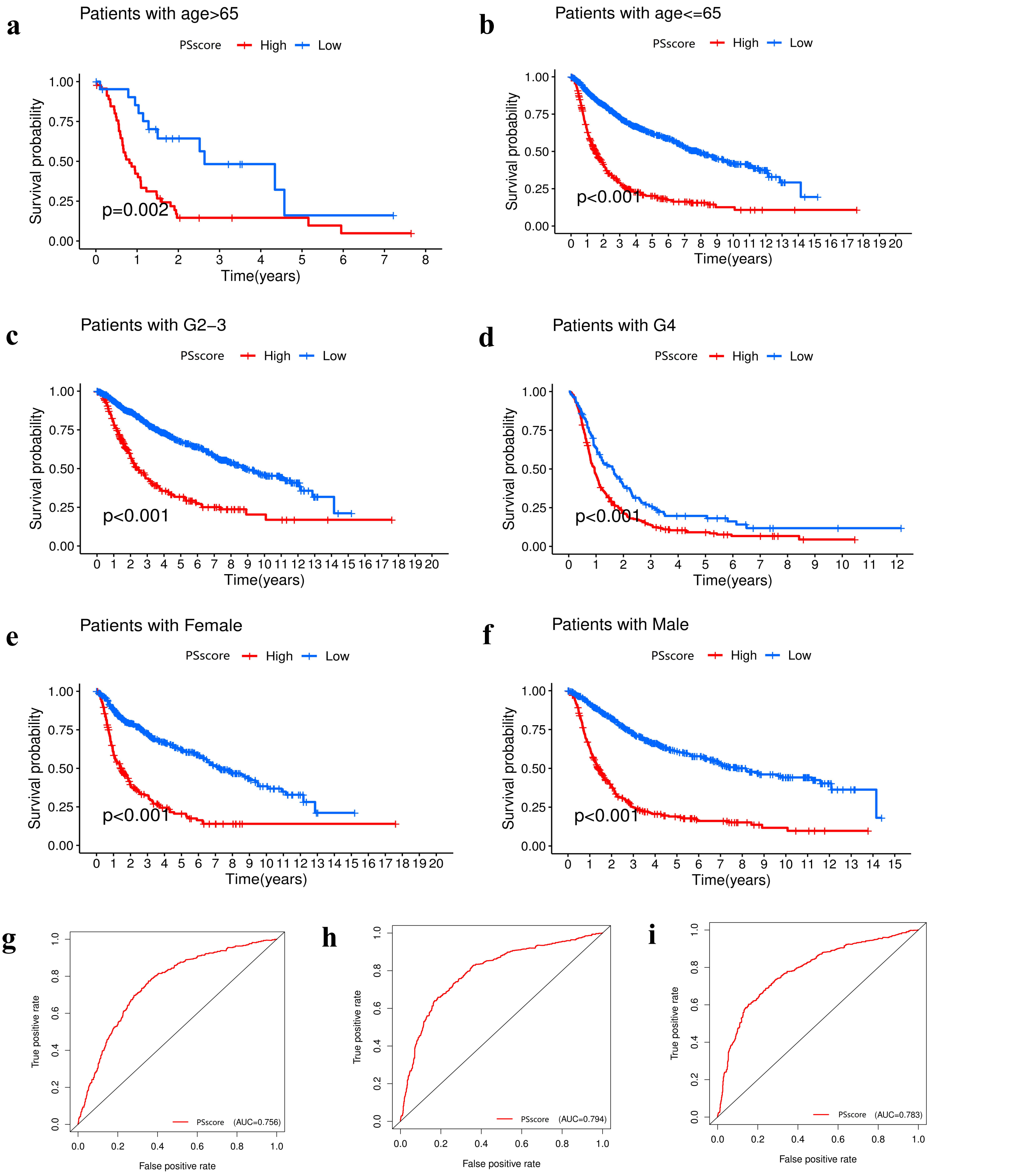

Fig. S6 OS of PSscore in glioma a-b. The survival curves in the different age groups were estimated by the Log-rank test (age>65, p=0.002; age<65, p<0.001). **c-d.** The survival curves in the different grade groups were estimated by the Log-rank test (Grade G2-3, p<0.001; Grade G4, p<0.001). **e-f.** The survival curves in the different gender groups were estimated by the Log-rank test (Male, p<0.001; Female, p<0.001). **g-i.** ROC curve analysis of the predictive value of PSscore in glioma cohorts (**g**, one-year, AUC 0.756; **h**, three-year, AUC 0.794; **i**, five-year, AUC 0.783).
